# 3-Hy­droxy-2-(hy­droxy­meth­yl)pyridinium chloride

**DOI:** 10.1107/S1600536811032405

**Published:** 2011-08-17

**Authors:** Richard Betz, Thomas Gerber, Eric Hosten

**Affiliations:** a Nelson Mandela Metropolitan University, Summerstrand Campus, Department of Chemistry, University Way, Summerstrand, PO Box 77000, Port Elizabeth 6031, South Africa

## Abstract

The cation of the title compound, C_6_H_8_NO_2_^+^·Cl^−^, is essentially planar (r.m.s. deviation = 0.0104 Å). Intermolecular O—H⋯Cl and N—H⋯Cl hydrogen bonds, as well as C—H⋯O contacts, connect the mol­ecules in the crystal structure. A short C⋯C distance of only 3.3930 (19) Å between C atoms of neighbouring rings is indicative of π-stacking. The corresponding centroid–centroid distance between the two aromatic systems is 4.2370 (7) Å due to the small overlap of the adjacent rings.

## Related literature

For the crystal structure of 3-hy­droxy-2-hy­droxy­methyl-6-methyl-pyridine, see: Casas *et al.* (2007 ▶). For graph-set analysis of hydrogen bonds, see: Etter *et al.* (1990 ▶); Bernstein *et al.* (1995 ▶). For general information about the chelate effect in coordination chemistry, see: Gade (1998 ▶).
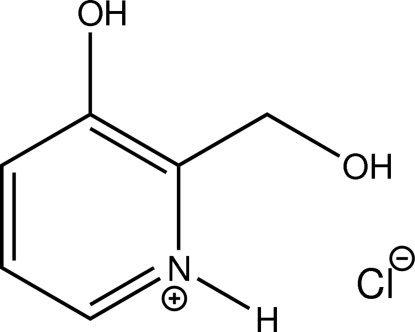


## Experimental

### 

#### Crystal data

C_6_H_8_NO_2_^+^·Cl^−^
*M* *_r_* = 161.58
Triclinic, 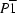

*a* = 6.8490 (2) Å
*b* = 7.1376 (2) Å
*c* = 7.9675 (2) Å
α = 73.895 (1)°
β = 68.634 (1)°
γ = 86.801 (1)°
*V* = 348.04 (2) Å^3^
*Z* = 2
Mo *K*α radiation
μ = 0.48 mm^−1^
*T* = 200 K
0.24 × 0.17 × 0.11 mm

#### Data collection

Bruker APEXII CCD diffractometer
Absorption correction: multi-scan (*SADABS*; Bruker, 2008 ▶) *T*_min_ = 0.834, *T*_max_ = 1.000
6143 measured reflections
1711 independent reflections
1572 reflections with *I* > 2σ(*I*)
*R*_int_ = 0.019

#### Refinement

*R*[*F* ^2^ > 2σ(*F* ^2^)] = 0.025
*wR*(*F* ^2^) = 0.073
*S* = 1.06
1711 reflections
103 parameters
H atoms treated by a mixture of independent and constrained refinement
Δρ_max_ = 0.35 e Å^−3^
Δρ_min_ = −0.17 e Å^−3^

### 

Data collection: *APEX2* (Bruker, 2010 ▶); cell refinement: *SAINT* (Bruker, 2010 ▶); data reduction: *SAINT*; program(s) used to solve structure: *SHELXS97* (Sheldrick, 2008 ▶); program(s) used to refine structure: *SHELXL97* (Sheldrick, 2008 ▶); molecular graphics: *ORTEP-3* (Farrugia, 1997 ▶) and *Mercury* (Macrae *et al.*, 2008 ▶); software used to prepare material for publication: *SHELXL97* and *PLATON* (Spek, 2009 ▶).

## Supplementary Material

Crystal structure: contains datablock(s) I, global. DOI: 10.1107/S1600536811032405/fy2019sup1.cif

Supplementary material file. DOI: 10.1107/S1600536811032405/fy2019Isup2.cdx

Structure factors: contains datablock(s) I. DOI: 10.1107/S1600536811032405/fy2019Isup3.hkl

Supplementary material file. DOI: 10.1107/S1600536811032405/fy2019Isup4.cml

Additional supplementary materials:  crystallographic information; 3D view; checkCIF report

## Figures and Tables

**Table 1:** Hydrogen-bond geometry (Å, °)

*D*—H⋯*A*	*D*—H	H⋯*A*	*D*⋯*A*	*D*—H⋯*A*
O1—H81⋯Cl1^i^	0.79 (2)	2.22 (2)	3.0086 (9)	176.1 (19)
O2—H82⋯Cl1^ii^	0.85 (2)	2.29 (2)	3.1276 (11)	168.7 (18)
N1—H71⋯Cl1^iii^	0.853 (17)	2.391 (17)	3.1739 (10)	152.9 (14)
C3—H3⋯O2^iv^	0.95	2.47	3.2069 (14)	134

Symmetry codes: (i) 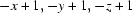; (ii) 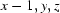; (iii) 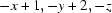; (iv) 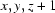.

**Fig. 1.**
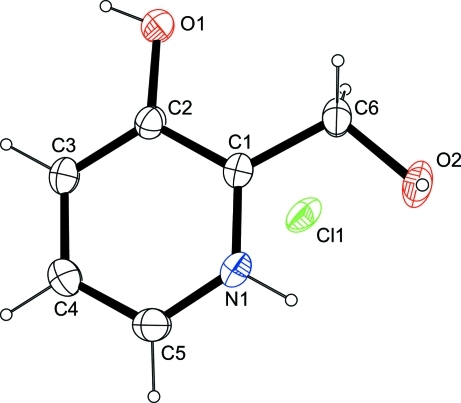
The molecular structure of the title compound, with atom labels and anisotropic displacement ellipsoids (drawn at 50% probability level).

**Fig. 2.**
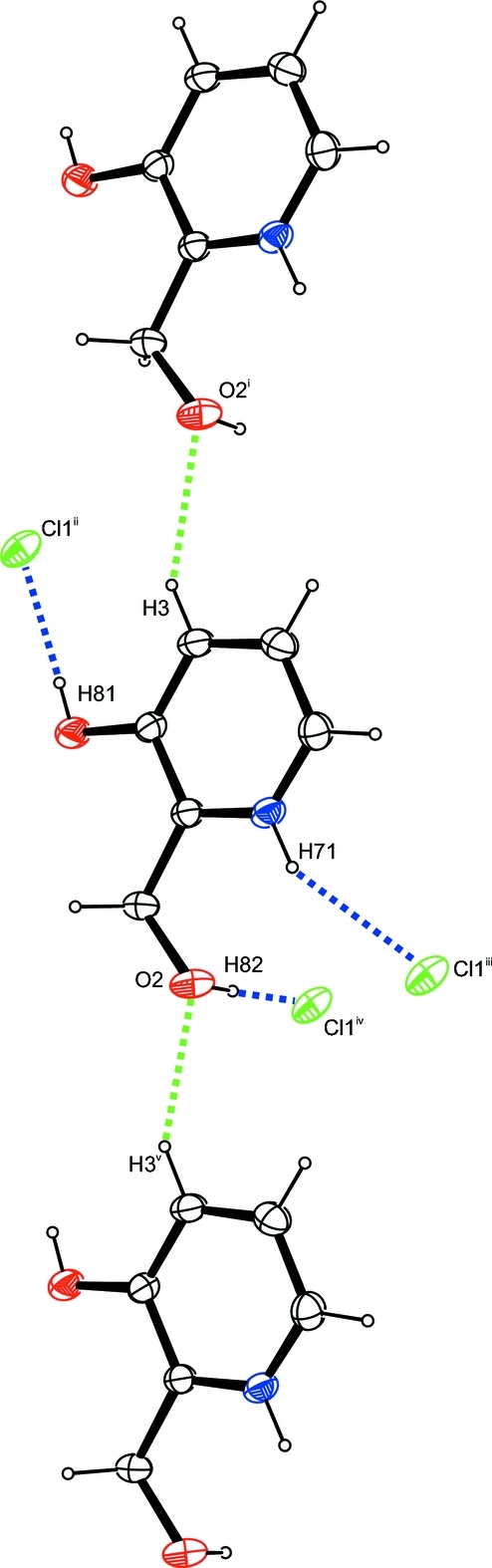
Intermolecular contacts, viewed approximately along [-1 -1 0]. Blue dashed lines indicate classical hydrogen bonds while green dashed lines indicate C–H···O contacts. Symmetry operators: (i) x, y, z + 1; (ii) -x + 1, -y + 1, -z + 1; (iii) -x + 1, -y + 2, -z; (iv) x - 1, y, z; (v) x, y, z - 1.

**Fig. 3.**
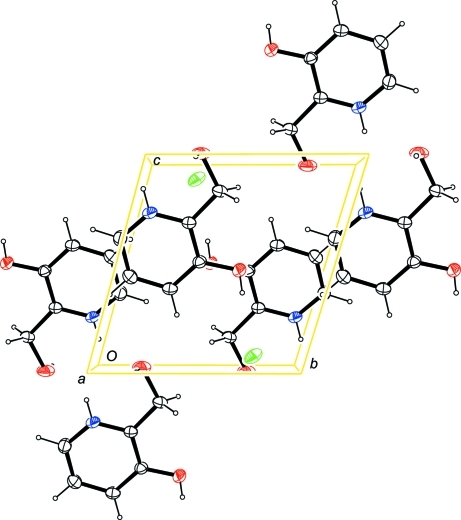
Molecular packing of the title compound, viewed along [-1 0 0] (anisotropic displacement ellipsoids drawn at 50% probability level).

## supplementary crystallographic information

### Comment

Chelate ligands have found widespread use in coordination chemistry due to the enhanced thermodynamic stability of resultant coordination compounds in relation to coordination compounds exclusively applying comparable monodentate ligands (Gade, 1998). Combining different donor atoms, a molecular set-up to accomodate a large variety of metal centers of variable Lewis acidity is at hand. In this aspect, the title compound seemed of interest due to its possible use as a strictly neutral or, depending on the pH value, as an anionic or cationic ligand. In addition, due to the set-up of its functional groups, it may act as mono- or bidentate ligand offering the possibility to create five- or six-membered chelate rings. To enable comparative studies in terms of bond lengths and angles in the envisioned coordination compounds, we determined the molecular and crystal structure of the title compound. Structural information about 3-hydroxy-2-hydroxymethyl-6-methyl-pyridine is available in the literature (Casas *et al.*, 2007).

Protonation took place on the nitrogen atom. Intracyclic angles span a range from 118.46 (10) ° to 123.99 (10) ° with the largest angle found on the nitrogen atom and the smallest angle on the carbon atom bearing the hydroxymethyl group. The non-hydrogen atoms of the organic cation essentially lie in one common plane (r.m.s. of fitted non-hydrogen atoms = 0.0104 Å). The hydroxymethyl group adopts a conformation in which the aliphatic hydroxyl group is bent away from the aromatic hydroxyl group (Fig. 1).

In the crystal structure, hydrogen bonds involving all hydroxyl groups and the protonated nitrogen atom as donors are present. In every case, the chloride anion serves as acceptor (Fig. 2). In addition, a C–H···O contact is observed whose range falls by more than 0.2 Å below the sum of van-der-Waals radii of the atoms participating. The latter contact involves the CH group in *ortho* position to the hydroxyl group bound to the aromatic system and the oxygen atom of the aliphatic hyxdroxyl group. A description of the classical hydrogen bonding system in terms of graph-set analysis (Etter *et al.*, 1990; Bernstein *et al.*, 1995) necessitates a *DDD* descriptor on the unitary level while the C–H···O contacts can be described by a *C*(6) descriptor at the same level. A C···C distance of only 3.3930 (19) Å between carbon atoms of neighbouring rings is indicative of π-stacking with the shortest intercentroid distance between two aromatic systems measured at 4.2370 (7) Å due to the small overlap of adjacent rings. In total, the components of the title compound are connected to a three-dimensional network with the C–H···O contacts forming chains along the crystallographic *c* axis.

The packing of the compound in the crystal structure is shown in Figure 3.

### Experimental

The compound was obtained commercially (Aldrich). Crystals suitable for the X-ray diffraction study were obtained upon slow evaporation of an aqueous solution of the compound at room temperature.

### Refinement

Carbon-bound H atoms were placed in calculated positions (C—H 0.95 Å for aromatic carbon atoms and C—H 0.99 Å for the methylene groups) and were included in the refinement in the riding model approximation, with *U*(H) set to 1.2*U*_eq_(C). The H atom of the hydroxyl groups as well as the H atom of the protonated nitrogen atom were located on a difference Fourier map and refined with individual thermal parameters.

### Crystal data

**Table:**

C_6_H_8_NO_2_^+^·Cl^−^	*Z* = 2
*M**_r_* = 161.58	*F*(000) = 168
Triclinic, *P*1	*D*_x_ = 1.542 Mg m^−^^3^
Hall symbol: -P 1	Mo *K*α radiation, λ = 0.71073 Å
*a* = 6.8490 (2) Å	Cell parameters from 4561 reflections
*b* = 7.1376 (2) Å	θ = 2.9–28.2°
*c* = 7.9675 (2) Å	µ = 0.48 mm^−^^1^
α = 73.895 (1)°	*T* = 200 K
β = 68.634 (1)°	Platelet, colourless
γ = 86.801 (1)°	0.24 × 0.17 × 0.11 mm
*V* = 348.04 (2) Å^3^	

### Data collection

**Table:**

Bruker APEXII CCD diffractometer	1711 independent reflections
Radiation source: fine-focus sealed tube	1572 reflections with *I* > 2σ(*I*)
graphite	*R*_int_ = 0.019
φ and ω scans	θ_max_ = 28.3°, θ_min_ = 2.9°
Absorption correction: multi-scan (*SADABS*; Bruker, 2008)	*h* = −9→9
*T*_min_ = 0.834, *T*_max_ = 1.000	*k* = −9→9
6143 measured reflections	*l* = −9→10

### Refinement

**Table:**

Refinement on *F*^2^	Primary atom site location: structure-invariant direct methods
Least-squares matrix: full	Secondary atom site location: difference Fourier map
*R*[*F*^2^ > 2σ(*F*^2^)] = 0.025	Hydrogen site location: inferred from neighbouring sites
*wR*(*F*^2^) = 0.073	H atoms treated by a mixture of independent and constrained refinement
*S* = 1.06	*w* = 1/[σ^2^(*F*_o_^2^) + (0.0395*P*)^2^ + 0.0944*P*] where *P* = (*F*_o_^2^ + 2*F*_c_^2^)/3
1711 reflections	(Δ/σ)_max_ = 0.001
103 parameters	Δρ_max_ = 0.35 e Å^−^^3^
0 restraints	Δρ_min_ = −0.17 e Å^−^^3^

### Fractional atomic coordinates and isotropic or equivalent isotropic displacement parameters (Å^2^)

**Table:**

	*x*	*y*	*z*	*U*_iso_*/*U*_eq_	
O1	0.25186 (15)	0.42693 (12)	0.51787 (12)	0.0286 (2)	
H81	0.240 (3)	0.382 (3)	0.624 (3)	0.047 (5)*	
O2	0.29516 (16)	0.75828 (14)	−0.01815 (11)	0.0325 (2)	
H82	0.166 (3)	0.776 (3)	0.001 (3)	0.051 (5)*	
N1	0.27167 (14)	0.91926 (14)	0.25202 (13)	0.0221 (2)	
H71	0.276 (2)	0.980 (2)	0.142 (2)	0.035 (4)*	
C1	0.26774 (16)	0.72440 (15)	0.29807 (14)	0.0197 (2)	
C2	0.25517 (16)	0.62326 (15)	0.47898 (15)	0.0205 (2)	
C3	0.24780 (18)	0.72716 (16)	0.60447 (15)	0.0243 (2)	
H3	0.2386	0.6601	0.7283	0.029*	
C4	0.25391 (18)	0.93031 (17)	0.54794 (16)	0.0263 (2)	
H4	0.2494	1.0026	0.6329	0.032*	
C5	0.26641 (18)	1.02541 (16)	0.36935 (16)	0.0258 (2)	
H5	0.2713	1.1640	0.3288	0.031*	
C6	0.28268 (19)	0.62276 (17)	0.15235 (15)	0.0247 (2)	
H6A	0.4087	0.5441	0.1314	0.030*	
H6B	0.1578	0.5329	0.1981	0.030*	
Cl1	0.81001 (5)	0.75715 (4)	0.07668 (4)	0.03225 (11)	

### Atomic displacement parameters (Å^2^)

**Table:**

	*U*^11^	*U*^22^	*U*^33^	*U*^12^	*U*^13^	*U*^23^
O1	0.0459 (5)	0.0196 (4)	0.0218 (4)	0.0029 (3)	−0.0161 (4)	−0.0034 (3)
O2	0.0412 (5)	0.0395 (5)	0.0163 (4)	0.0019 (4)	−0.0116 (4)	−0.0050 (3)
N1	0.0253 (5)	0.0228 (4)	0.0162 (4)	0.0008 (3)	−0.0081 (3)	−0.0013 (3)
C1	0.0195 (5)	0.0229 (5)	0.0166 (5)	0.0015 (4)	−0.0069 (4)	−0.0049 (4)
C2	0.0221 (5)	0.0210 (5)	0.0183 (5)	0.0018 (4)	−0.0086 (4)	−0.0038 (4)
C3	0.0299 (5)	0.0269 (5)	0.0181 (5)	0.0016 (4)	−0.0118 (4)	−0.0054 (4)
C4	0.0305 (6)	0.0271 (5)	0.0253 (5)	0.0010 (4)	−0.0119 (4)	−0.0111 (4)
C5	0.0292 (5)	0.0202 (5)	0.0279 (6)	0.0005 (4)	−0.0108 (4)	−0.0056 (4)
C6	0.0306 (6)	0.0278 (5)	0.0173 (5)	0.0025 (4)	−0.0096 (4)	−0.0074 (4)
Cl1	0.03770 (18)	0.02940 (16)	0.02636 (16)	−0.00094 (11)	−0.01668 (13)	0.00473 (11)

### Geometric parameters (Å, °)

**Table:**

O1—C2	1.3482 (13)	C2—C3	1.3866 (15)
O1—H81	0.79 (2)	C3—C4	1.3921 (16)
O2—C6	1.4085 (13)	C3—H3	0.9500
O2—H82	0.85 (2)	C4—C5	1.3692 (16)
N1—C1	1.3355 (14)	C4—H4	0.9500
N1—C5	1.3470 (15)	C5—H5	0.9500
N1—H71	0.853 (17)	C6—H6A	0.9900
C1—C2	1.3948 (14)	C6—H6B	0.9900
C1—C6	1.5025 (14)		
			
?···?	?		
			
C2—O1—H81	108.9 (14)	C4—C3—H3	120.2
C6—O2—H82	100.9 (13)	C5—C4—C3	119.70 (10)
C1—N1—C5	123.99 (10)	C5—C4—H4	120.1
C1—N1—H71	118.0 (11)	C3—C4—H4	120.1
C5—N1—H71	118.0 (11)	N1—C5—C4	118.92 (10)
N1—C1—C2	118.46 (10)	N1—C5—H5	120.5
N1—C1—C6	119.00 (9)	C4—C5—H5	120.5
C2—C1—C6	122.52 (10)	O2—C6—C1	111.08 (9)
O1—C2—C3	124.70 (10)	O2—C6—H6A	109.4
O1—C2—C1	115.98 (9)	C1—C6—H6A	109.4
C3—C2—C1	119.32 (10)	O2—C6—H6B	109.4
C2—C3—C4	119.61 (10)	C1—C6—H6B	109.4
C2—C3—H3	120.2	H6A—C6—H6B	108.0
			
C5—N1—C1—C2	0.71 (16)	C1—C2—C3—C4	−0.27 (17)
C5—N1—C1—C6	−177.70 (10)	C2—C3—C4—C5	0.23 (18)
N1—C1—C2—O1	−179.96 (9)	C1—N1—C5—C4	−0.76 (17)
C6—C1—C2—O1	−1.61 (15)	C3—C4—C5—N1	0.26 (17)
N1—C1—C2—C3	−0.18 (15)	N1—C1—C6—O2	−1.18 (14)
C6—C1—C2—C3	178.17 (10)	C2—C1—C6—O2	−179.52 (10)
O1—C2—C3—C4	179.49 (11)		

### Hydrogen-bond geometry (Å, °)

**Table:**

*D*—H···*A*	*D*—H	H···*A*	*D*···*A*	*D*—H···*A*
O1—H81···Cl1^i^	0.79 (2)	2.22 (2)	3.0086 (9)	176.1 (19)
O2—H82···Cl1^ii^	0.85 (2)	2.29 (2)	3.1276 (11)	168.7 (18)
N1—H71···Cl1^iii^	0.853 (17)	2.391 (17)	3.1739 (10)	152.9 (14)
C3—H3···O2^iv^	0.95	2.47	3.2069 (14)	134.

Symmetry codes: (i) −*x*+1, −*y*+1, −*z*+1; (ii) *x*−1, *y*, *z*; (iii) −*x*+1, −*y*+2, −*z*; (iv) *x*, *y*, *z*+1.
